# DNA-PKcs and PARP1 at the interface between DNA damage responses and cGAS–STING signaling: context-dependent roles and therapeutic implications

**DOI:** 10.1080/15384047.2026.2728335

**Published:** 2026-09-08

**Authors:** Tiantian Miao, Jiaqi Zhao, Jinghong Wu, Jiabao Hou, Teng Ma, Guirong Wang

**Affiliations:** a Department of Clinical Laboratory, Beijing Chest Hospital, Capital Medical University, Beijing Tuberculosis and Thoracic Tumor Institute, Beijing, People's Republic of China; b Cancer Research Center, Beijing Chest Hospital, Capital Medical University, Beijing Tuberculosis and Thoracic Tumor Research Institute, Beijing, People's Republic of China

**Keywords:** DNA-PKcs, PARP1, cGAS–STING, NHEJ, BER, cancer

## Abstract

**Aims:**

To explore the roles of DNA-dependent protein kinase catalytic subunit (DNA-PKcs) and poly(ADP-ribose) polymerase 1 (PARP1) in both the DNA damage repair (DDR) pathway and the cyclic GMP–AMP synthase–stimulator of interferon genes (cGAS–STING) pathway mediated immune response, and to analyze the therapeutic potential of their inhibitors.

**Methods:**

This is a review article synthesizing recent findings on the functions of DNA-PKcs and PARP1 in DDR, their context-dependent effects on the cGAS–STING pathway and the therapeutic mechanisms of their inhibitors.

**Results:**

DNA-PKcs and PARP1 are key components of two major DDR mechanisms. Beyond their canonical repair functions, both factors significantly regulate the cGAS–STING pathway, a central mediator linking cytoplasmic DNA and the type I interferon response.

**Conclusion:**

DNA-PKcs and PARP1 connect genome maintenance with innate immune signaling through context-dependent mechanisms. Targeting these proteins represents a promising strategy for modulating cGAS–STING signaling and improving disease treatment.

## Graphical abstract

**Figure:**
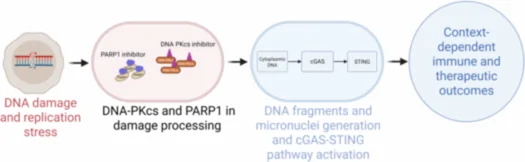

DNA-PKcs and PARP1 inhibitors may exert antitumor effects by impairing DNA repair and, in appropriate contexts, promoting cytosolic DNA accumulation and cGAS–STING activation.

## 1. Introduction

Human cells experience approximately 70,000 DNA damage events daily. These events include DNA double-strand breaks (DSBs), DNA single-strand breaks (SSBs), and nucleotide alterations such as deletions, insertions, and substitutions. To preserve genomic stability, cells activate multiple DNA damage response (DDR) pathways, including base excision repair (BER), nucleotide excision repair (NER), mismatch repair (MMR), translesion DNA synthesis (TLS), non-homologous end joining (NHEJ), and homologous recombination (HR).^1^ Among these pathways, DNA-dependent protein kinase catalytic subunit (DNA-PKcs) is a core component of NHEJ-mediated DSB repair, whereas poly(ADP-ribose) polymerase 1 (PARP1) plays a key role in single-strand break repair (SSBR) and facilitates BER by sensing and coordinating the repair of SSB intermediates generated during the processing of oxidative DNA lesions. Although these proteins are canonically associated with distinct DNA repair pathways, accumulating evidence indicates that both DNA-PKcs and PARP1 participate in the recognition and repair of complex DNA damage. The Ku heterodimer (Ku70/Ku80) recognizes sites of DNA damage and recruits DNA-PKcs to form the DNA-PK complex, which phosphorylates both the DNA-PKcs and downstream repair proteins to coordinate repair.

PARP1 binds directly to SSBs and uses nicotinamide adenine dinucleotide(NAD+) as a substrate to catalyze the poly(ADP-ribosyl)ation (PARylation) of itself and other repair factors, thereby facilitating SSBR. Because DNA-PKcs and PARP1 have overlapping functions in the repair of complex lesions and the maintenance of genomic integrity, both proteins have attracted interest as therapeutic targets in cancer and other human diseases.^2^ ^,^ ^3^

The cGAS–STING pathway is a central mediator of innate immune responses to misplaced or cytosolic DNA. Currently, its role in tumor biology is attracting widespread attention because the activation of this pathway can promote senescence in precancerous cells, mediate apoptosis, and enhance the host antitumor immunity. Both exogenous DNA from microorganisms (bacteria, viruses, protozoa) and endogenous DNA generated during cellular stress can activate the cGAS–STING pathway.^4^ cGAS recognizes cytosolic DNA, catalyzes the synthesis of cyclic GMP–AMP (cGAMP), thereby triggering a series of cascade reactions that ultimately leads to the production of type I interferons and other cytokines.^5^ The cGAS–STING pathway has been implicated in malignant tumors, inflammatory disorders, and autoimmune diseases.

Evidence indicates that DNA-PKcs and PARP1 modulate cGAS–STING signaling through distinct, context-dependent mechanisms. DNA-PK can restrain cGAS activity by phosphorylating cGAS and limiting its ability to bind DNA.^6^ Inhibition of PARP1 promotes the accumulation of dsDNA and enhances activation of the cGAS–STING pathway.^7^ Furthermore, DNA-PKcs and PARP1 inhibitors, alone or in combination, can enhance antitumor immunity in vivo through this pathway. To date, many studies have identified the therapeutic potential of targeting these two DDR factors via cGAS–STING signaling. Therefore, a critical synthesis of these findings may inform future mechanistic studies and clinical translation.

In this review, we aim to highlight the roles of DNA-PKcs and PARP1 in DDR and the cGAS–STING pathway. We explored the therapeutic potential of inhibiting these DDR factors, either alone or in combination, in cancer, autoimmune disorders, and viral infections. We also proposed a biological model in which DNA damage, DDR signaling, and innate immune activation are interconnected.

## 2. The roles and interactions of DNA-PKcs and PARP1 in DDR

### 2.1. The structure of DNA-PKcs and its role in DDR

**Figure 1.**
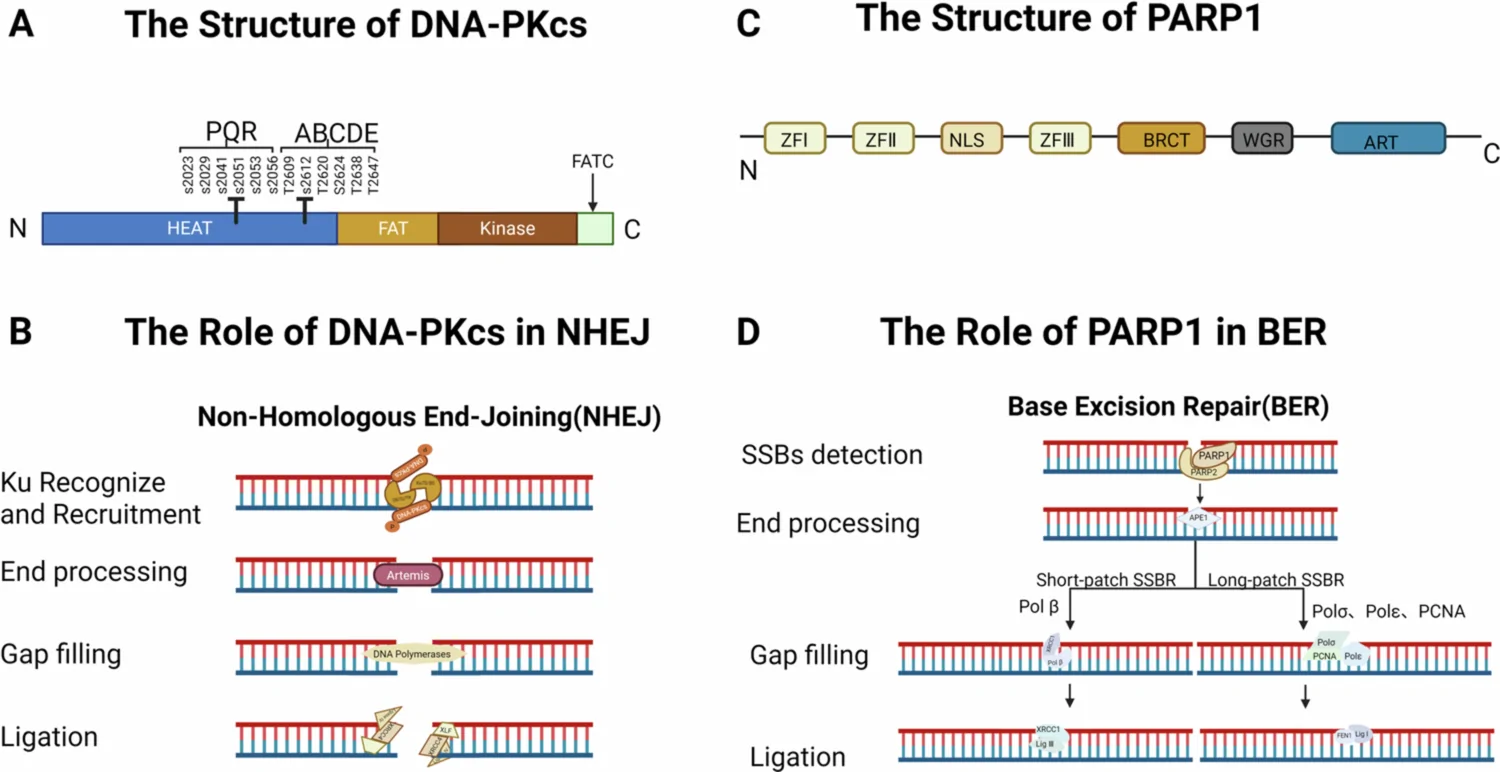
Principal roles of DNA-PKcs and PARP1 in DDR. (A) DNA-PKcs contains four principal domains. (B) DNA-PKcs primarily functions in NHEJ. (C) PARP1 contains four principal domains. (D) PARP1 primarily functions in BER. The figure was created with BioRender.com.

DNA-PKcs is a member of the phosphoinositide 3-kinase (PI3K)-related kinase (PIKK) family given its structural similarity to other PIKKs. Members of this family share conserved HEAT (Huntingtin, Elongation Factor 3, PP2A, and TOR1) repeats, FAT/FATC (FRAP, ATM, TRAPP/C-terminus), FRB(FKBP-rapamycin-binding), and kinase domains (Figure 1). The highly conserved kinase domain confers the catalytic activity of DNA-PKcs. And the HEAT domain is also crucial for the overall function and activation state of DNA-PKcs. The FATC region contains sites that regulate DNA-PKcs activity, and conformational changes in the FAT domain can modulate kinase activity.^8^

Cells primarily repair DSBs by HR or classical non-homologous end joining (C-NHEJ). C-NHEJ repairs DSBs via blunt-end ligation and requires the coordinated action of factors such as Ku70/80, DNA-PKcs, and DNA ligase IV.^9^ The Ku70/80 heterodimer and the catalytic subunit DNA-PKcs constitute DNA-PK.^10^ Upon DSBs, the Ku heterodimer rapidly recognizes the break ends and recruits DNA-PKcs. The Ku then undergoes an inward translocation, positioning DNA-PKcs at the break ends.^11^ ^,^ ^12^ DNA-PKcs undergoes auto-phosphorylation at Thr2609 in a Ku-dependent manner. The phosphorylated protein co-localizes with *γ*H2AX and 53BP1 proteins near DSBs.^13^ Artemis is activated through physical contact with autophosphorylated DNA-PKcs.^14^ DNA-PK autophosphorylation triggers a conformational change that reorients the DNA, allowing Artemis to recognize and cleave ssDNA–dsDNA junctions.^15^ ^,^ ^16^ After Artemis trims the DNA ends, the DNA ligase IV complex completes ligation. DNA-PKcs can recognize different types of DNA ends, forming either “protective” or “processing” complexes to promote trans-phosphorylation of other targets or cis-autophosphorylation of ABCDE.^17^ Recruitment of the XRCC4–ligase IV complex to DNA ends and activation of ligase IV require DNA-PK. XRCC4 is also phosphorylated at DNA ends, and this phosphorylation depends on DNA-PKcs.^18^ ^,^ ^19^

DNA-PKcs also regulates HR. It phosphorylates replication protein A (RPA), promoting dissociation of the RPA-p53 complex, allowing RPA to bind more effectively to ssDNA, and promoting HR via RAD51 recombinase (RAD51).^20^ The phosphorylation state of DNA-PKcs remains a key factor influencing repair-pathway choice. During replication stress, DNA-PK is the principal kinase responsible for phosphorylation of RPA32 at Ser4/Ser8, an early event required for full ATR activation and establishment of the replication checkpoint.^21^ However, the role of DNA-PKcs in the switching between NHEJ and HR repair pathways under replicative stress remains unclear.

DNA-PKcs also contributes to the oxidative stress response and redox homeostasis. H₂O₂ induces excessive activation of the ATM signaling pathway and reduces cell survival in cells lacking DNA-PKcs. Furthermore, the deficiency of DNA-PKcs leads to increased production of reactive oxygen species (ROS). These findings reveal a non-canonical, NHEJ-independent function of DNA-PKcs in counteracting oxidative stress.^22^ DNA-PKcs also participates in the processing of complex and clustered DNA lesions. Clustered DNA lesions include double-stranded breaks (DSBs) and non-DSB oxidatively-induced clustered DNA lesions (OCDLs), which are repaired inefficiently when DNA-PKcs is absent. This may be because DNA-PKcs inhibition leads to the persistence of DSBs and prevents DNA-PKcs from dissociating from DNA ends. The presence of a DSB or the DNA-PK molecule inhibits the processing of adjacent clustered DNA lesions.^23^ Furthermore, DNA-PK inhibition has been shown to lead to the accumulation of DNA damage (manifested as micronuclei) induced by the chemotherapeutic drug bleomycin.^24^ These unresolved clusters of damage can lead to replication stress and micronuclei, which may further trigger immune activation in the body.

### 2.2. The structure of PARP1 and its role in DDR

The poly(ADP‐ribose) polymerase (PARP) family comprises nicotinamide adenine dinucleotide (NAD⁺)‐dependent enzymes that catalyze ADP‐ribosylation and regulate multiple physiological processes. So far, seventeen PARP family members have been identified in humans. PARP1 contains four domains: *N*-terminal DNA-binding domain (DBD), BRCT domain, WGR domain, and C-terminal catalytic ART domain.^25^ The *N*-terminal DBD of PARP1 comprises three zinc fingers (ZFI, ZFII and ZFIII), a nuclear localization signal (NLS) and a caspase-3 cleavage site.^26^ ^,^ ^27^ The *N*-terminal zinc fingers of PARP-1 can convert the SSB of DNA into a highly folded DNA conformation, drive SSB sensing, stimulate the interaction with the scaffold protein XRCC1, and promote subsequent DNA repair.^28^ ZFII and ZFIII are essential for recognizing and binding to DNA damage sites, while the NLS directs PARP1 to the nucleus. ZFIII participates in enzyme activation after DNA binding, playing a vital role in DNA-dependent stimulation of PARP1. Caspase-3 cleaves PARP1 within its DBD and reduces its catalytic activities.^27^ ^,^ ^29^ The WGR domain contains a tryptophan (W)-glycine (G)-arginine (R) motif and helps regulate DNA-dependent catalytic activity.^30^ Zn1, Zn2 and the WGR domain sense DNA damage and connect the DNA-damage interface to the catalytic domain. DNA initiation triggers allosteric signaling, while Zn^2^⁺ and NAD⁺ enhancement modifies signaling patterns and strengthens activation interfaces. At this point, the conformation of PARP1 destabilizes the CAT, promoting PARP1 DNA-dependent activity.^31^ ^,^ ^32^

After binding an SSB or DSB, PARP1 becomes catalytically active and uses NAD⁺ to attach ADP-ribose (ADPR) to itself and other nuclear proteins.^33^ PARP1 can catalyze the generation of free ADPR and free poly(ADP-ribose) (PAR) molecules that are not attached to proteins.^34^ ^,^ ^35^

One of the best-characterized DNA-repair functions of PARP1 is its role as a sensor and coordinator of SSBR. SSBs can arise directly or as intermediates during base excision repair (BER).^36^ Upon binding to SSBs, PARP1 PARylates itself and nearby proteins and releases free PAR. XRCC1 and DNA ligase IIIα are recruited to the damage site via BRCT domains binding to PAR.^37^ PARP1 plays a dual role in the BER pathway: as an active player and as a decelerator.^38^ ^,^ ^39^ Researchers have also discovered that PARP1 participates in the repair of a specific type of ssDNA nick generated when DNA topoisomerase 1 (TOP1) activity is aborted. PARP1 recruits and activates tyrosyl-DNA phosphodiesterase 1 (TDP1) to eliminate abortive TOP1–DNA complexes, also known as TOP1 cleavage complexes (TOP1cc).^40^ ^,^ ^41^

PARP contributes to DSB repair by mediating chromatin relaxation, promoting signaling pathways related to DSBs, recruiting various chromatin remodeling factors, and regulating the selection of repair pathways.^42^ PARP1 participates in three DSB repair pathways—HR, c-NHEJ, and alternative non-homologous end joining (alt-NHEJ). PARylated BRCA1 interacts with RAP80, limiting strand invasion and subsequent HR repair.^43^ In c-NHEJ pathway, PARP1 recruits XRCC4 and the chromatin remodeler CHD2.^44^ In alt-NHEJ, PARP1 recruits MRE11 to process DNA ends.^45^ ^,^ ^46^ PARP1 can also activate microhomology-mediated Alt-EJ (MMEJ).^47^

PARP1 also functions in both major branches of NER. In global genome nucleotide excision repair (GG-NER), PARP1 binds to damage-binding protein 2 (DDB2) at UV-damaged chromatin and enhances its catalytic activity.^48^ PARP1 interacts with CSB on the lesion-stalled RNA polymerase II and stabilizes the CSB–RNA polymerase II complex, thereby promoting the initiation of transcription-coupled nucleotide excision repair (TCR).^49^

When the excision of the dsDNA ends at replication forks is hindered, PARP and DNA-PK bind to the unexcised ends and recruit XRCC1 protein, enabling the replication fork to continue excision and restart replication.^50^

Beyond lesion repair, PARP1 plays a crucial role in processes such as DNA methylation, chromatin remodeling, and transcriptional activation. It can oppose DNA methylation by inhibiting the catalytic activity of DNA methyltransferases and promoting the expression of ten–eleven translocation dioxygenases involved in demethylation.^51^ ^,^ ^52^ Researchers have discovered that PARP inhibitors enhance supraphysiological androgen (SPA)-induced cellular senescence and DNA damage in prostate cancer cells.^53^

Thus, PARP1 is active not only in DNA repair but also in transcriptional regulation, immune signaling, and metabolism. Defining how these functions vary across tumor and non-tumor settings will be important for understanding the therapeutic benefits and toxicities of PARP-directed interventions.^54^

### 2.3. The interactions between DNA-PKcs and PARP1 in DDR

PARP1 and DNA-PK can bind similar DNA structures in vitro and can also modify one another. DNA-PK can phosphorylate PARP without affecting PARP’s catalytic activity or DNA-binding ability, while PARP can ADP-ribosylate DNA-PKcs and activate it independently of the Ku70/80 complex.^55^ Moreover, PARylation of DNA-PKcs prevents sustained activation, whereas PARP1 inhibitors prevent DNA-PKcs dissociation from chromatin. However, this is a potential mechanism, and PARP inhibitors still exert antitumor effects.^56^ In CNE-2 cells exposed to VP-16, shRNA was used to knock down the DNA-PKcs and PARP1 genes. Knockdown of PARP1 reduces both mRNA and protein levels of DNA-PKcs, while knockdown of DNA-PKcs downregulates PARP1 and PAR expression.^57^

The interactions between DNA-PKcs and PARP1 in DDR are regulated by multiple factors. Banf1 inhibits PARP1 auto-ADP-ribosylation and also binds to and inhibits DNA-PKcs, thereby altering the repair-pathway choice.^58^ Trp-tRNA synthetase (TrpRS) can bridge DNA-PKcs and PARP1 in a ternary complex. The kinase domain of DNA-PKcs and the N-terminal domain of PARP-1 are connected via the WHEP domain of TrpRS, thereby promoting PARylation of DNA-PKcs and downregulating the phosphorylation of PARP1. This plays a crucial role in linking IFN-*γ* and p53 signaling pathways.^59^ GCN5 undergoes PARylation by PARP1, thereby being recruited to DSBs, where it supports activation of NHEJ kinases. DNA-PKcs is acetylated by GCN5 at the K3241 site, a prerequisite for its S2056 phosphorylation and recruitment to DNA damage sites. Additionally, GCN5 regulates DNA-PKcs transcription.^60^ Similarly, ZNF432 enhances PARP1 activation and influences the dynamics of PARylation at DNA damage sites; PAR production in turn facilitates ZNF432 recruitment, whereas ZNF432 inhibition reduces phosphorylated DNA-PKcs.^61^ Androgen receptor (AR) signaling also regulates PARP1 and DNA-PKcs expression. AR inhibition lowers both proteins, and PARP1 inhibition reduces AR, DNA-PKcs, and γH2AX levels, consistent with an AR–PARP1 positive-feedback loop in DDR.^62^

However, the interaction between PARP1 and DNA-PKcs is not uniformly protective and can also promote adverse cellular outcomes. PARP1 recognizes ssDNA gaps amplified by MRE11, driving the initial activation of DNA-PKcs and further establishing the signaling program that drives replication catastrophe—irreversible replication-fork collapse caused by excessive ssDNA accumulation.^63^ Researchers discovered that the interaction between PARP and DNA-PKcs leads to 14-3-3σ-induced radioresistance: 14-3-3σ promotes PARP1 expression, which in turn facilitates the recruitment of DNA-PKcs to DNA damage sites.^64^ These findings underscore that the biological consequences of DNA-PKcs–PARP1 crosstalk depend on the type and extent of damage and on the cellular context.

## 3. DNA-PKcs in cGAS–STING research

**Figure 2.**
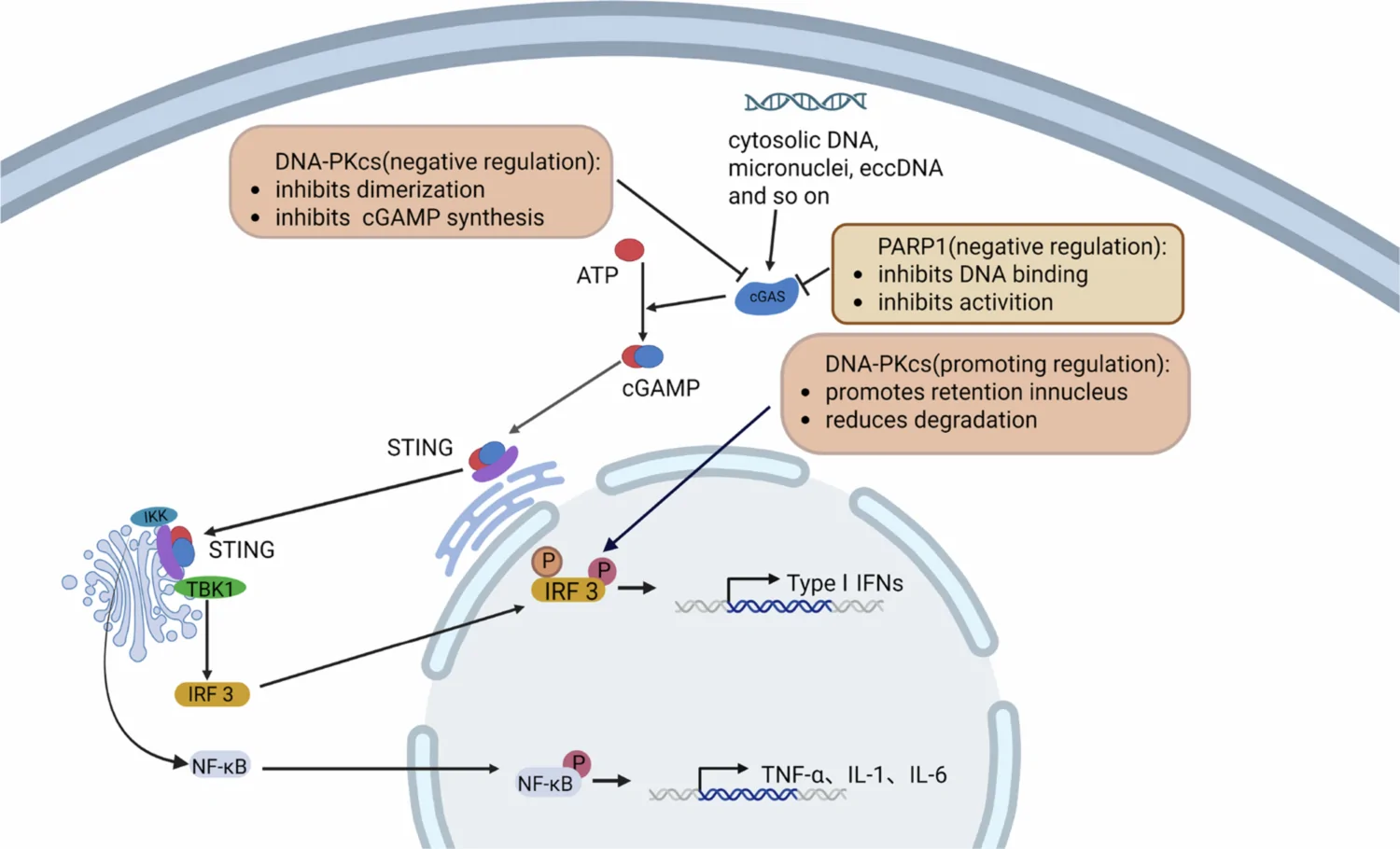
Overview of cGAS–STING signaling and its modulation by DNA-PKcs and PARP1. The figure was created with BioRender.com.

Upon binding to DNA, cGAS undergoes a conformational change to its active state and synthesizes the second messenger cGAMP (Figure 2). cGAMP binds to and activates the cyclic-dinucleotide sensor STING. STING subsequently translocates to the Golgi apparatus, where it first binds to and activates TANK-binding kinase 1 (TBK1). Subsequently, it recruits and activates interferon regulatory factor 3 (IRF3). Phosphorylated IRF3 dimerizes and enters the nucleus, initiating the expression of type I interferons and other inflammatory and chemokine genes.^65^ ^,^ ^66^ STING also recruits the IκB kinase (IKK), thereby releasing nuclear factor κB(NF-κB) to activate pro-inflammatory cytokine genes (such as TNF and IL-6).^67^ TBK1 regulates dsDNA-mediated NF-κB activation signaling.^68^ cGAS is primarily localized within the cell nucleus, while cytoplasmic cGAS is mainly found in the inner leaflet of the plasma membrane. The STING protein has diverse functions, playing a crucial role not only in the cGAS–STING pathway but also in the activation of the NF-κB pathway.^69^ Ultimately, it upregulates the expression of type I interferons (IFNs), interferon-stimulated genes (ISGs), and senescence-associated secretory phenotypes (SASPs). Furthermore, STING mediates autophagy through a mechanism that remains incompletely understood.^70^ The cGAS–STING axis exhibits dual roles in antitumor immunity. On one hand, it induces type I interferon synthesis, promotes dendritic cells (DCs) maturation and antigen presentation, enhances natural killer (NK) cells proliferation and IFN-*γ* production capacity, recruits and activates CD8+ T cells, thereby exerting antitumor effects. On the other hand, this axis can upregulate programmed cell death Protein 1 (PD-1) and programmed cell death ligand 1 (PD-L1) expression, recruit myeloid-derived suppressor cells (MDSCs), drive T-cell exhaustion, and facilitate tumor immune escape.^71-73^, This pathway not only plays a role in the host's defense against DNA viruses, retroviruses, bacteria, and parasites. It also serves as a key mediator of functional decline during aging and chronic inflammation. In addition, the cGAS–STING pathway plays a role in certain autoimmune diseases, such as coatomer subunit *α* syndrome, which is a prominent STING-associated autoimmune disease.^74^

The sources of DNA that activate cGAS include not only DNA viruses, retroviruses, and intracellular bacteria, but also stressed mitochondrial DNA (mtDNA) and chromatin fragments arising from mitotic errors, replication stress or DNA damage.^65^ Cytoplasmic DNA generated by DNA damage, such as oxidative stress, radiation, chromosomal instability, excessive activation of oncogene signaling pathways, and chemotherapies can activate the cGAS–STING pathway.^70^ Defects in DDR lead to the formation of micronuclei. Rupture of the micronuclear envelope exposes chromatin to cGAS and activates the pathway.^75^ ^,^ ^76^ Furthermore, extrachromosomal circular DNA (eccDNA) generated by DNA damage repair, replication stress, and chromothripsis also activates the STING innate immune pathway via the cGAS sensor.^77^ Together, these processes provide a mechanistic continuum from lesion complexity and failed repair to replication stress, cytosolic DNA generation, and innate immune activation.

DNA-PK has dual and context-dependent roles in the cGAS–STING pathway. First of all, DNA-PK acts as a negative regulator in the cGAS–STING pathway. As a negative regulator, DNA-PK can phosphorylate cGAS: phosphorylation at Thr68 interferes with cGAS dimerization, whereas phosphorylation at Ser213 suppresses cGAS activation and cGAMP synthesis. DNA-PK-mediated phosphorylation of cGAS serves as a checkpoint that maintains immune homeostasis. The deficiency of DNA-PK enhances cGAS-mediated innate immune responses, leading to increased expression of downstream IFNB1 and CXCL10.^78^ Cytoplasmic cGAS acts as an immune sensor, whereas nuclear cGAS influences repair-pathway choice. The cGAS–Ku80 complex stabilizes DNA-PKcs through USP7-mediated deubiquitination, thereby favoring c-NHEJ over alternative NHEJ.^79^ By contrast, DNA-PK plays a promoting role in the immune pathway during viral infection. DNA-PK-mediated phosphorylation of IRF3 at Thr135 promotes its nuclear retention and limits its degradation, thereby supporting IFN transcription.^80^ Moreover, cytoplasmic DNA-PK plays an immunostimulatory role. Acting as a DNA sensor, it relies on IRF3, TBK1, and STING to trigger the transcription of downstream type I IFN, cytokine, and chemokine genes.^10^

At the signaling level, the DNA-PK complex has been described as an IRF3-activating DNA sensor, a component of a human STING-independent DNA-sensing pathway, and a facilitator of cGAS–STING signaling.^10^ Conversely, DNA-PK-mediated phosphorylation of cGAS can suppress its catalytic activity.^78^ These apparently divergent findings likely reflect differences in species, cell type, DNA source, subcellular context, and experimental perturbation. DNA-PKcs should therefore be viewed as a context-dependent regulator that both restrains immunostimulatory DNA generation through DNA repair and modulates downstream sensing after DNA becomes accessible to cytosolic sensors.

## 4. PARP1 in cGAS–STING research

PARP1 also acts as a negative regulator in the cGAS–STING pathway. First of all, PARP1 can interact with cGAS. cGAS inhibits both PARP1-mediated PAR synthesis in the presence of double-stranded DNA and DNA-RNA hybrids. cGAS binds to PAR via its zinc finger domain, thereby inhibiting PARP1 activation.^81^ Within the nucleus, cGAS not only impedes the interaction between PARP1 and Timeless but also inhibits HR.^82^ DNA damage caused by viral infection activates DNA-PK, which phosphorylates PARP1 at the Thr594 site and promotes its translocation to the cytoplasm. Cytoplasmic PARP1 then PARylates cGAS at position D191, inhibiting further DNA binding and activation of cGAS.^83^ Furthermore, inhibiting PARP1 UFMylation impairs its function, leading to DNA damage and R-loop formation, thereby activating the cGAS–STING pathway.^84^ STING is also closely associated with PARP1. PARP1 overexpression has also been associated with fewer cGAS- and STING-positive cells, reduced CD8+ T-cell infiltration, increased regulatory T-cell infiltration, and lower cytokine secretion, whereas PARP1 inhibition produces antitumor effects in the reported model.^85^ STING can increase PARP1 activity, PAR release, and NAD⁺ and ATP consumption, regulating the death of CD4+ T cells.^86^ STING inhibition suppresses PARP1 expression, and STING exacerbates inflammatory lung injury in sepsis through PARP1-related pathways.^87^

The regulation of the PARP1-mediated STING pathway is subject to multiple factors. Researchers have discovered that Src homology-2 domain-containing protein tyrosine phosphatase-2 (SHP2) dephosphorylates PARP1 and inhibits its activity, thereby increasing DNA damage and promoting the activation of the cGAS–STING pathway.^88^ Pseudorabies virus (PRV) exploits an opposing mechanism: PRV infection promotes non-muscle myosin heavy chain-IIA (NMHC-IIA) expression, thereby enhancing the interaction between PARP1 and cGAS. This reduces cGAS binding to DNA, and suppresses the antiviral innate immune response mediated by the cGAS–STING pathway.^89^ Lysine 254 of cGAS undergoes SIRT3-mediated decrotonylation under ionizing radiation exposure, reducing the cGAS-DNA binding complex and inhibiting cGAS-PARP1 interaction. This alleviates HR suppression, indicating that inhibiting SIRT3 enhances cancer cell sensitivity to ionizing radiation. These observations suggest that inhibiting SIRT3 may sensitize cancer cells to radiation by altering cGAS–PARP1 signaling.^90^

At the direct signaling level, cytoplasmic PARP1 can PARylate human cGAS and reduce its DNA-binding capacity in an antiviral context.^83^ PARP1 can also support NF-κB-dependent transcription through a non-catalytic coactivator function.^91^ The immunological functions of PARP1 are bidirectional and context-dependent.

## 5. DNA-PK inhibitors and PARP inhibitors in cGAS–STING research

### 5.1. DNA-PK inhibitors in cGAS–STING research

Building upon the regulatory roles of DNA-PKcs in the cGAS–STING pathway discussed above, increasing evidence suggests that DNA-PKcs inhibitors can modulate innate immune signaling and enhance antitumor immunity through activation of the cGAS–STING pathway. In many models, this effect appears to arise indirectly from unrepaired DNA damage and the generation of cytosolic DNA.

The combined therapy of DNA-PKcs inhibitor NU7441 and DNA-damaging inducer doxorubicin is closely associated with the cGAS–STING pathway. Inhibition of DNA-PKcs impairs DNA double-strand break (DSB) repair and increases cytoplasmic DNA, which is sensed by cGAS. Moreover, the combination enhanced the cGAS–STING-dependent type I IFN response and increased expression of related genes. DNA-PKcs inhibition also reduced cGAS binding to histones, potentially increasing its availability to bind cytosolic DNA. The combination promoted cGAS–STING-dependent apoptosis and M1 macrophage polarization, thereby limiting the tumor-supportive activity of M2 macrophages (Table 1).^92^ DNA-PKcs rapidly phosphorylates the C-terminal tail of H2AX at Ser139. The resulting γH2AX helps recruit repair proteins to DSBs.^93^ ^,^ ^94^ Phosphorylated H2AX at the S139 site interacts with cGAS, promoting the recruitment of cGAS to sites of DNA damage.^82^ Furthermore, combining ionizing radiation with NU7441 or PRKDC gene interference increased γH2AX and dsDNA. γH2AX promoted cGAS recruitment, whereas cGAS sensing of dsDNA was accompanied by phosphorylation of downstream TBK1 and IRF3 and activation of cGAS–STING signaling.^95^ Similarly, the DNA-PKcs inhibitor M3814 enhanced radiation-induced killing of cancer cells, induced micronucleus formation and promoted activation of the cGAS-STING inflammatory pathway.^96^

Collectively, these findings suggest that DNA-PKcs inhibitors can enhance cGAS–STING signaling by increasing unrepaired DNA, promoting cytosolic DNA and micronucleus formation, and facilitating cGAS recruitment. This indirect sequence provides a rationale for combining DNA-PKcs inhibitors with radiotherapy or other DNA-damaging agents, although its therapeutic value is likely to depend on tumor context and immune competence.

### 5.2. PARP inhibitors in cGAS–STING research

PARP inhibitors can suppress catalytic activity and, to varying degrees, trap PARP1 on DNA. The resulting replication-associated damage can generate cytosolic dsDNA that is recognized by cGAS, thereby activating cGAS–STING signaling. The requirement for PARP1 in this response supports a contribution from PARP1 trapping rather than catalytic inhibition alone.^97^

The PARP inhibitor olaparib induces apoptosis and suppresses colony formation in BCR/ABL1-positive cells, potentially in part through cGAS–STING activation and increased interferon expression. These findings support its therapeutic potential in BCR/ABL1-driven leukemia.^98^ In multiple myeloma co-culture models, talazoparib induced immunogenic cell death; this effect was lost after cGAS knockout, indicating dependence on cGAS–STING signaling.^99^ In cisplatin-resistant gastric cancer cells, the PARP1 inhibitors AG14361 and BYK204165 suppressed DNA-PKcs and increased cisplatin-induced DNA damage and apoptosis.^100^

Niraparib activates cGAS–STING signaling and increases CCL5 and CXCL10. These changes are accompanied by PD-L1 upregulation and CD8+ T-cell infiltration, providing a rationale for combining niraparib with PD-L1 blockade in ovarian cancer.^101^ Olaparib can likewise increase tumor-cell PD-L1 through cGAS–STING signaling. In the LLC tumor model, platinum, olaparib, and PD-L1 blockade showed antitumor activity, while olaparib-induced DNA damage increased cytosolic genomic DNA and pathway activation.^102^ These findings support combination strategies involving PARP inhibition, chemotherapy, and immune-checkpoint blockade. Olaparib plus trabectedin increased TBK1 and IRF3 phosphorylation to activate cGAS–STING pathway and the expression of ligands that activate NK and cytokine-induced killer (CIK) cells. The activated lymphocytes killed tumor cells and counteracted persistent OCT4-positive cells, suggesting potential utility in advanced sarcoma.^103^ In pancreatic cancer models, disulfiram combined with chemotherapy reduced PARP1 expression, increased cytosolic dsDNA, and activated cGAS–STING signaling.^104^ The combination of the PARP inhibitor olaparib and a STING agonist enhances STING pathway activation, boosts interferon signaling and antigen processing, and strengthens the function of cytotoxic T cells and DCs. This combination demonstrates superior efficacy compared to monotherapy in BRCA1-deficient breast cancer models.^105^ Olaparib plus CDK12-IN-3 increased nuclear cGAS and nearly abolished cGAS Tyr215 phosphorylation. These changes suppressed HR and increased genomic instability in HR-proficient ovarian cancer cells.^106^ In ERCC1-deficient cells, olaparib or rucaparib generated cytoplasmic chromatin fragments (CCFs), activated cGAS–STING signaling, increased type I IFN expression, and stimulated CCL5 secretion. PARP inhibition plus IFN-*γ* also increased PD-L1 expression.^107^ ^,^ ^108^ By contrast, ERCC1-proficient cells efficiently excised PARP1-associated lesions and repaired DSBs by HR, producing too few CCFs to activate the pathway. This contrast illustrates how repair capacity determines whether PARP inhibition is converted into an innate immune signal.

Liu et al. engineered a novel PARP inhibitor from Olaparib, termed the B3 compound, which potently dual-inhibits PARP1 and PARP7. This disrupts DDR, activates the cGAS–STING pathway, enhances CD8+ T cell infiltration, and achieves antitumor effects.^109^ The PARP1 inhibitor 4F-DDC induces DNA damage and cGAS–STING activation and suppresses the growth of HCC-1937-derived xenografts.^110^ CN0 (compound 3) degrades PARP1 via the proteasome, thereby activating the cGAS–STING pathway. This compound represents a promising therapeutic approach capable of overcoming PARP inhibitor resistance.^111^ Compound 10n, a dual PARP/NAMPT inhibitor, activates the cGAS–STING pathway, promotes immunogenic cell death and the release of damage-associated molecular patterns (DAMPs), enhances T-cell killing, and suppresses the mobilization of MDSCs in BRCA-wild-type triple-negative breast cancer models.^112^ PN-Ola encapsulates olaparib within a PD-L1-targeting amphiphilic peptide-photosensitizer conjugate C16-K(PpIX)-WHRSYYTWNLNT. By inhibiting DDR and activating cGAS–STING signaling, it increases CD4+ and CD8+ T-cell infiltration; the associated increase in PD-L1 may further enhance tumor targeting.^113^ A stimuli-responsive and dual PROTAC-embedded immunoactivator (denoted as Sd@Lip), containing the PARP1 degrader SK-575 and the BRD4 degrader dBET1, was developed to inhibit NHEJ and HR simultaneously. It enhanced STING activation, increased IFN-*β* and other pro-inflammatory mediators, and promoted CD8+ T-cell and NK-cell infiltration.^114^ Although promising, these agents remain preclinical and require comparative evaluation of selectivity, toxicity, and dependence on an intact cGAS–STING pathway.

Beyond cancer, olaparib can also activate antiviral innate immunity through the cGAS–STING pathway and prevent PRV viral infection.^115^ The PARP1–cGAS pathway has also been investigated as a therapeutic target for chronic inflammation in Chagas cardiomyopathy caused by Trypanosoma cruzi.^116^

This diverse mechanisms provide a strong rationale for combining PARP inhibitors with immunotherapy, chemotherapy, and other targeted therapies.

### 5.3. Combination therapy of DNA-PKcs inhibitors and PARP inhibitors for cGAS–STING research

Combining the DNA-PKcs inhibitor AZD7648 with PARP inhibitors suppresses tumor-cell growth, increases apoptosis, and induces tumor regression in preclinical models.^117^ Olaparib plus NU7441 simultaneously impairs HR and NHEJ and may therefore be useful in hepatocellular carcinoma (HCC). Mechanistically, PARP1 supports HR by recruiting ALC1 to damage sites and promoting chromatin relaxation; PARP1 inhibition consequently reduces RPA2 and RAD51 recruitment, whereas DNA-PKcs inhibition compromises NHEJ.^118^ NU7441 and olaparib also sensitized nasopharyngeal carcinoma (NPC) cells to VP-16.^57^ In addition, NU7441 and olaparib enhanced responses to radiotherapy in HPV-negative head and neck squamous cell carcinoma, and AZD7648, talazoparib, and niraparib radiosensitized head and neck squamous cell carcinoma (HNSCC) cell lines in vitro.^119^ ^,^ ^120^ Notably, beyond inhibiting tumors through the classical DNA repair pathways described above, DNA-PK and PARP1 inhibitors can also suppress rRNA synthesis and translation, thereby inhibiting the growth of BRCA-deficient tumor cells without generating a significant increase in DSBs.^121^ However, the impact of this combination on cGAS–STING signaling remains incompletely understood and warrants further investigation.

As shown in Table 2, the clinical trials involving DNA-PKcs inhibitors or PARP1 inhibitors are summarized.

**Table 1.** DNA-PKcs inhibitors/PARP1 inhibitors combination therapies and applications.

DNA-PKcs inhibitor/PARP1 inhibitor	Combination therapy	Diseases
NU7441	Doxorubicin; IR	MM^92^ ; HNSCC^95^
M3814	Radiation and Bintrafusp alfa	Locally advanced solid tumors^96,96^
Olaparib	Platinum and PD-L1 blodcade; Trabectedin; STING agonism(ADU-S100); CDK12-IN-3	Lung adenocarcinoma^102^ ; Advanced sarcomas^103^ ; BRCA1-deficient breast cancer^105^ ; HR-proficient ovarian cancer^106^ ; BCR/ABL1-mediated leukemia^98^; PRV viral infection^115^
DSF	Gemcitabine, Anti-PD 1 antibody	Pancreatic cancer^104^
Olaparib or Rucaparib		ERCC1-deficient non–small cell lung cancer^107^
Niraparib	PD-L1 Inhibitors	Ovarian cancer^101^
AG14361 or BYK204165	Cisplatin	Cisplatin-resistant gastric cancer^100^
Talazoparib		Multiple myeloma^99^
4F-DDC		Breast cancer^110^
Compound 10n		BRCA wild-type triple-negative breast cancer^112^
PN-Ola		Metastatic tumor^113^
NU7441 and Olaparib	Radiotherapy	HCC^118^ ; Nasopharyngeal carcinoma ^57^ ; HPV-negative squamous cell carcinoma of the head and neck^119^

**Table 2.** Clinical trials related to DNA-PK inhibitors or PARP1 inhibitors.

NCT number	Study status	Conditions	Interventions	Key Objective
NCT02516813	COMPLETED	Advanced Solid Tumors	DRUG: M3814 RADIATION: Fractionated RT Drug: Cisplatin	evaluate the safety, the tolerability and the efficacy
NCT02316197	COMPLETED	Advanced Solid Tumors|Chronic Lymphocytic Leukemia	DRUG: MSC2490484A (M3814)	determine the highest dose and the appropriate dose
NCT05002140	COMPLETED	Metastasis|Locally Advanced Solid Tumor|Recurrent Cancer	DRUG: XRD-0394 RADIATION: Palliative radiotherapy	evaluate the safety and tolerability
NCT06253130	RECRUITING	Advanced Solid Tumor	DRUG: IMP1734	evaluate the safety, tolerability and preliminary efficacy
NCT00687765	COMPLETED	Glioblastoma	DRUG: BSI-201 plus Temozolomide	determine the Maximum Tolerated Dose (MTD)
NCT01082549	COMPLETED	Squamous Cell Lung Cancer	DRUG: Gemcitabine/Carboplatin DRUG: Gemcitabine/Carboplatin plus Iniparib	evaluate the overall survival (OS) of patients
NCT06931626	RECRUITING	Small Cell Lung Cancer	DRUG: NMS-03305293 Drug: Temozolomide	determine the safety and tolerability, as well as to evaluate the anti-tumor efficacy and pharmacokinetics
NCT06930755	RECRUITING	Ovarian Cancer	DRUG: NMS-03305293 DRUG: Topotecan	determine the safety and tolerability, as well as to evaluate the anti-tumor efficacy and pharmacokinetics
NCT06907043	RECRUITING	Advanced Solid Tumors	DRUG: EIK1004-001 (IMP1707-001)	evaluate the safety, tolerability, and preliminary efficacy
NCT01983358	COMPLETED	Stroke	Drug: JPI-289 OTHER: Placebo	evaluate safety, tolerability, pharmacokinetics
NCT01086254	COMPLETED	NSCLC Stage IV	DRUG: Iniparib DRUG: Gemcitabine DRUG: Cisplatin	assess the objective response rate (ORR)
NCT00920595	COMPLETED	Solid Tumor	DRUG: CEP-9722	evaluate the safety, pharmacokinetics, and pharmacodynamics
NCT00516373	COMPLETED	Ovarian Neoplasms|BRCA1 Protein|BRCA2 Protein	DRUG: KU-0059436 (AZD2281)(PARP inhibitor)	determine the safety, tolerability, dose-limiting toxicity (DLT), pharmacokinetic-pharmacodynamic profile, and MTD
NCT06421935	ACTIVE_NOT_RECRUITING	Advanced Solid Tumor	DRUG: M9466|DRUG: Tuvusertib|DRUG: Abiraterone acetate|DRUG: Prednisone/Prednisolone	evaluate the safety, tolerability, pharmacokinetic, pharmacodynamic profile
NCT01204125	COMPLETED	Breast Cancer Female	DRUG: Paclitaxel|DRUG: Iniparib (SAR2405550 -BSI-201)	assess the pathological Complete Response (pCR) rate
NCT01045304	COMPLETED	Breast Cancer, Metastatic	DRUG: Iniparib|DRUG: Gemcitabine|DRUG: Carboplatin	assess the ORR
NCT00664781	COMPLETED	BRCA1 Mutation Carrier|BRCA2 Mutation Carrier|Breast Cancer|Ovarian Cancer	DRUG: Rucaparib (CO-338; formally AG-014699 or PF-01367338)	study the side effects and best dose
NCT01345357	COMPLETED	Solid Tumors or Mantle Cell Lymphoma	DRUG: CEP-9722|DRUG: Gemcitabine|DRUG: Cisplatin	determine MTD
NCT01540565	COMPLETED	BRCA1 Mutation Carrier|BRCA2 Mutation Carrier|Ovarian Epithelial Tumor|Recurrent Fallopian Tube Carcinoma|Recurrent Ovarian Carcinoma|Recurrent Primary Peritoneal Carcinoma	DRUG: Veliparib	investigate Efficacy

## 6. Research gaps and key controversies

DNA-PKcs and PARP1 inhibitors, alone or in combination, have shown antitumor activity in preclinical studies. However, whether combined inhibition produces additive, synergistic, or qualitatively distinct effects on cGAS–STING signaling remains unclear. Before clinical use, toxicity, safety, and any potential carcinogenic effects must be evaluated rigorously. Studies on mechanisms and disease applications are not sufficiently in-depth. Future in-depth exploration of the combination therapy for disease treatment and its underlying mechanisms is crucial.

PARP inhibitors act through two related but distinct mechanisms. First, catalytic inhibition blocks PARP-mediated PARylation, impairs SSB repair, and is particularly effective in tumors with HR defects such as BRCA1 or BRCA2 mutations. Second, some inhibitors trap PARP proteins on DNA and thereby obstruct replication and transcription. Trapped PARP–DNA complexes can be more cytotoxic than unrepaired SSBs produced by catalytic inactivation alone. Trapping is promoted by loss of PARP1 automodification because the negatively charged ADP-ribose chains on automodified PARP1 normally facilitate its release from DNA through electrostatic repulsion.^122–124^ Olaparib, niraparib, rucaparib, and talazoparib have been approved for selected BRCA-mutated breast, ovarian, prostate, and pancreatic cancers. Nevertheless, resistance remains a major limitation. Many patients acquire resistance during prolonged treatment, and in fact, PARP inhibitor has limited efficacy in over 40% of BRCA1/2 mutation patients.^125^ Developing agents that overcome resistance is therefore a major priority. Thioparib has shown activity in resistant models, but further validation is needed.^126^ Research indicates that 10 triazole derivatives exhibit superior binding affinity compared to the reference PARP1 inhibitor, positioning them as potential PARP1 inhibitors.^127^ The clinical translation of DDRi first requires addressing the issue of drug resistance, which includes primary resistance (e.g., tumors with normal HR activity showing limited response to PARPi) and acquired resistance (e.g., BRCA-mutated tumors regaining DNA repair capacity through the restoration of mutations or compensatory pathways). There is even potential cross-resistance among different DDRi.^128^

## 7. Conclusion: prospects for their combined effects

**Figure 3.**
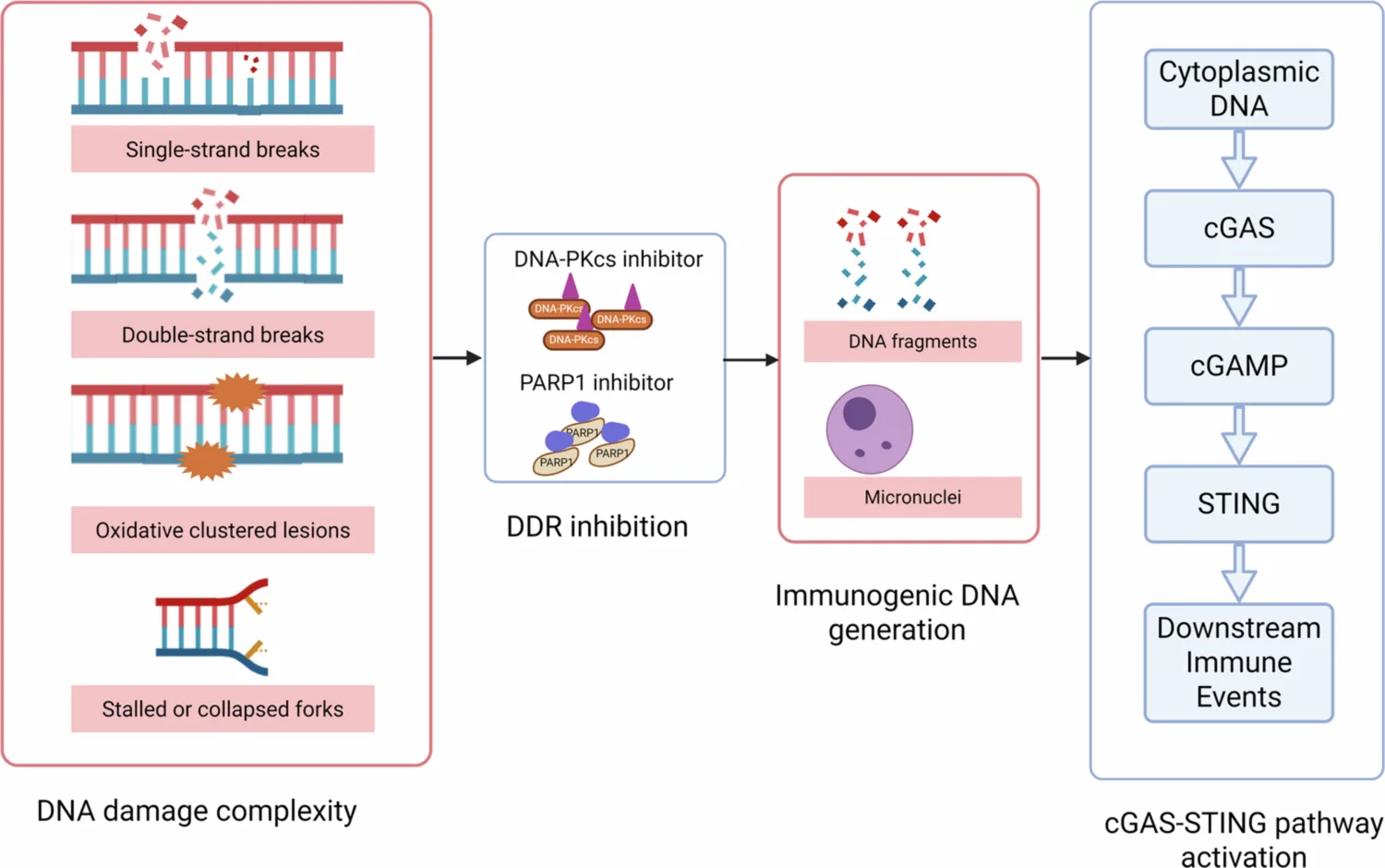
A unified biological model integrating different forms of DNA damage, DDR signaling, and innate immune activation. The figure was created with BioRender.com.

This review has examined the functions of DNA-PKcs and PARP1 in DDR and cGAS–STING signaling and has evaluated the therapeutic potential of targeting these proteins. This study proposes a biological model (Figure 3). Base lesions and single-strand breaks (SSBs) are normally processed by base excision repair and single-strand break repair, with PARP1 acting as a sensor and coordinator of strand-break intermediates, whereas exposed double-strand break (DSB) ends prominently engage DNA-PKcs-dependent non-homologous end joining. However, these categories converge during S phase: unrepaired base lesions, SSB intermediates, clustered oxidative lesions, and PARP1 trapping can impede replication-fork progression. The disruption of replication fork integrity is one of the primary causes of DNA accumulation in the cytoplasm following replicative stress, and the inactivation of DNA repair factors further exacerbates the accumulation of nuclear DNA in the cytoplasm. The cytoplasmic DNA originates from the ssDNA, dsDNA, and micronuclei generated by this process. Some of these structures may expose self-DNA in a form permissive for cGAS activation, thereby engaging STING–TBK1–IRF3 and NF-κB signaling. This transition is further gated by the duration and cellular context of signaling.^129^

Currently, targeting the DDR offers new insights for cancer immunotherapy. Combination therapy with DDRi and immunotherapy may be more effective in BRCA1/2-associated tumors (such as breast cancer, ovarian cancer, and prostate cancer) than in BRCA1/2-unrelated tumors (such as small cell lung cancer and gastric cancer). Tumors with homologous recombination deficiency (HRD) or BRCAness features are most likely to benefit from DDR–STING targeting strategies. DDR-targeted therapies enhance tumor immunogenicity and reshape the tumor immune microenvironment. Increasing genomic instability can enhance immunotherapy. Defects in HR, BER, or NER pathways are associated with elevated tumor mutation burden (TMB) and neoantigen load (NAL), and tumors with high TMB often develop an inflammatory microenvironment, thereby enhancing the efficacy of ICIs. By utilizing TMB and NAL as biomarkers and strategically targeting DDR pathways, personalized tumor immunotherapy can be achieved.^128^

Beyond pharmacological inhibition, emerging gene-editing technologies may provide alternative approaches for targeting DDR pathways. In recent years, gene editing has gained attention in the treatment of multiple cancers. BRCA1/2 mutations often predict the efficacy of PARP inhibitors in ovarian cancer, breast cancer, prostate cancer, and other cancers.^129^ A rapid functional assay based on CRISPR-Cas9 genome editing can assess BRCA1/2 variants, enabling PARP inhibitor therapy for individuals with BRCA1/2 dysfunction.^130^

ABL1 kinase inhibitors enhance the sensitivity of AML1-ETO and NUP98-PMX1-induced leukemia cells to DNA-PKcs inhibitors.^131^ These findings suggest that combination therapies involving DNA-PKcs inhibitors, PARP1 inhibitors, and their sensitizers deserve further investigation.

We need to identify more biomarkers to predict resistance and treatment efficacy of DNA-PKcs and PARP inhibitors. For example, tumor-derived IL-34 induces PARP inhibitor resistance and serves as a poor prognostic factor in ovarian serous carcinoma.^132^ The DDR–Immune Fitness (DDR-IF) score integrates homologous recombination deficiency, tumor mutational burden, and STING pathway activity to identify patients with extensive-stage small-cell lung cancer who may benefit from PARP inhibitor–immune-checkpoint blockade. Its clinical utility, however, requires confirmation in prospective multicenter studies.^133^ Micronuclei are another potential biomarker because they can activate cGAS–STING signaling and promote inflammation. Whether their abundance predicts individual sensitivity to immunotherapy remains unknown.

The efficacy, safety, and underlying mechanisms of combination therapy with PARP inhibitors and immune checkpoint inhibitors (CTLA-4 inhibitors, PD-1/PD-L1 inhibitors) have been investigated in various solid tumors. The combination of other DDRi (such as CHK1 inhibitors, WEE1 inhibitors, and DNA-PK inhibitors) with ICIs is also a current research focus in the field of cancer immunotherapy. Several DDRi monotherapies (such as CHK1 inhibitors, WEE1 inhibitors, and DNA-PK inhibitors) have achieved success in clinical trials, providing valuable insights for the future design of combination strategies and treatment optimization.^128^

In summary, DNA-PKcs and PARP1 connect the response to complex DNA damage with cGAS–STING-dependent immunity through multiple direct and indirect mechanisms. Therapeutic targeting is promising, but successful translation will require context-specific biomarkers, a clear distinction between catalytic inhibition and protein trapping, and careful management of immune activation and toxicity.

## Data Availability

All data are available in the main text.

## Abbreviations

DDR: DNA damage response
BER: Base excision repair
NER: Nucleotide excision repair
MMR: Mismatch repair
TLS: Translesion DNA synthesis
NHEJ: Non-homologous end joining
HR: Homologous recombination
DNA-PKcs: DNA-dependent protein kinase catalytic subunit
DSBs: Double-strand breaks
SSBs: Single-strand breaks
Ku70/80: Ku heterodimer (Ku70/Ku80 complex)
DNA-PK: DNA-dependent protein kinase complex
PARP1: Poly(ADP-ribose) polymerase 1
NAD⁺: Nicotinamide adenine dinucleotide
PARylation: Poly(ADP-ribosyl)ation
cGAS–STING: cyclic GMP–AMP synthase–stimulator of interferon genes pathway
cGAS: cyclic GMP–AMP synthase
cGAMP: cyclic GMP–AMP
STING: Stimulator of interferon genes
TBK1: TANK-binding kinase 1
IRF3: Interferon regulatory factor 3
IFN: Interferon
NF-κB: Nuclear factor κB
ISGs: Interferon-stimulated genes
SASPs: Senescence-associated secretory phenotypes
PD-1: Programmed cell death protein 1
PD-L1: Programmed cell death ligand 1
MDSCs: Myeloid-derived suppressor cells
NK cells: Natural killer cells
DCs: Dendritic cells
ATM: Ataxia telangiectasia mutated
ATR: ATM and Rad3-related
RPA: Replication protein A
RAD51: RAD51 recombinase
XRCC1: X-ray repair cross-complementing protein 1
XRCC4: X-ray repair cross-complementing protein 4
DNA ligase IV: DNA ligase IV
EPHA2: Ephrin type-A receptor 2
ROS: Reactive oxygen species
γH2AX: phosphorylated histone H2AX (Ser139)
TOP1: Topoisomerase I
TOP1cc: TOP1 cleavage complexes
PAR: Poly(ADP-ribose)
PARP: Poly(ADP-ribose) polymerase family
BRCT: BRCA1 C-terminal
WGR: Trp-Gly-Arg
DBD: DNA-binding domain
ZF: Zinc finger
NLS: Nuclear localization signal
OCDLs: Oxidatively induced clustered DNA lesions
ssDNA: single-stranded DNA
dsDNA: double-stranded DNA
HRD: Homologous recombination deficiency
TMB: Tumor mutational burden
NAL: Neoantigen load
ICIs: Immune checkpoint inhibitors
CTLA-4: Cytotoxic T-lymphocyte-associated protein 4
IL-6: Interleukin-6
TNF: Tumor necrosis factor
CXCL10: C-X-C motif chemokine ligand 10
CCL5: C-C motif chemokine ligand 5
MMEJ: Microhomology-mediated end joining
Alt-EJ: Alternative end joining
Alt-NHEJ: alternative non-homologous end joining
CCFs: Cytoplasmic chromatin fragments
PRV: Pseudorabies virus
EGFR-AS1: EGFR antisense RNA 1
NMNAT1: Nicotinamide mononucleotide adenylyltransferase 1
TCR: Transcription-coupled nucleotide excision repair
GG-NER: Global genome nucleotide excision repair
DAMPs: Damage-associated molecular patterns
SPA: Supraphysiological androgen
MRE11: Meiotic recombination 11 homolog
CHD2: Chromodomain helicase DNA-binding protein 2
ALC1: Amplified in liver cancer 1
Treg: Regulatory T cells
CIK: Cytokine-induced killer cells
IR: Ionizing radiation
HNSCC: Head and neck squamous cell carcinoma
NPC: Nasopharyngeal carcinoma
HCC: Hepatocellular carcinoma
NSCLC: Non-small cell lung cancer
AML: Acute myeloid leukemia
SSBR: Single-strand break repair
